# Targeting the two-pore channel 2 in cancer progression and metastasis

**DOI:** 10.37349/etat.2022.00072

**Published:** 2022-02-28

**Authors:** Kathryn A. Skelding, Daniel L. Barry, Danielle Z. Theron, Lisa F. Lincz

**Affiliations:** 1 Cancer Cell Biology Research Group, School of Biomedical Sciences and Pharmacy, College of Health, Medicine and Wellbeing, The University of Newcastle, Callaghan, New South Wales 2308, Australia; 2 Hunter Medical Research Institute, New Lambton Heights, New South Wales 2305, Australia; 3 Hunter Hematology Research Group, Calvary Mater Newcastle Hospital, Waratah, New South Wales 2298, Australia; The University of Texas at Arlington, USA

**Keywords:** Two-pore channel 2, TPCN2, tetrandrine, naringenin, cancer, anti-cancer drugs, verapamil

## Abstract

The importance of Ca^2+^ signaling, and particularly Ca^2+^ channels, in key events of cancer cell function such as proliferation, metastasis, autophagy and angiogenesis, has recently begun to be appreciated. Of particular note are two-pore channels (TPCs), a group of recently identified Ca^2+^-channels, located within the endolysosomal system. TPC2 has recently emerged as an intracellular ion channel of significant pathophysiological relevance, specifically in cancer, and interest in its role as an anti-cancer drug target has begun to be explored. Herein, an overview of the cancer-related functions of TPC2 and a discussion of its potential as a target for therapeutic intervention, including a summary of clinical trials examining the TPC2 inhibitors, naringenin, tetrandrine, and verapamil for the treatment of various cancers is provided.

## Introduction

Ca^2+^ is a major second messenger in cells and alterations in intracellular Ca^2+^ signaling regulate a variety of biological processes, including exocytosis/endocytosis, cell proliferation, invasion, migration, and apoptosis. Dysregulation of Ca^2+^ signaling is increasingly being demonstrated to contribute to the development of cancer [1], and has been implicated in each of the hallmarks of cancer originally identified by Hanahan and Weinberg [2].

For the most part, alterations in intracellular Ca^2+^ occur due to the opening of Ca^2+^-channels. Many different types of Ca^2+^-channels are found in cells; some are expressed on the plasma membrane [e.g., voltage-gated Ca^2+^ channels, transient receptor potential (TRP) channels, and store-operated channels], whereas others are expressed on the membrane of intracellular organelles, such as ryanodine receptors (RyRs) and inositol 1,4,5-trisphosphate receptors (IP_3_R) located on the sarcoplasmic and endoplasmic reticulum. Two-pore channels (TPCs) are unique in that they are located on the acidic organelles of the endolysosomal system [3].

The endolysosomal system is a complex pathway of dynamic organelles responsible for the delivery of cargo from the cell surface to internal lysosomes via trafficking through early endosomes and late endosomes. Final transfer to lysosomes is achieved by the fusion of late endosomes with lysosomes to form transient hybrid organelles known as endolysosomes, from which lysosomes are eventually reformed. These acidic, protease-laden organelles were referred to as “suicide bags” by the scientist who discovered them [4], making them attractive targets for cancer therapies that could permeabilize their membranes to allow toxic hydrolases to escape into the cytosol. But it is now known that the endosomal network is used for a multitude of cellular functions, including recycling and cell signaling, cell death and survival, and ultimately maintaining cell homoeostasis [5]. Thus, the seemingly simple concept of lysosome membrane destabilization as a therapeutic option has taken on more complexity, but also offers more potential.

The recently identified TPC2 (also known as TPCN2) Ca^2+^-channel provides the seemingly perfect anti-cancer target. Although the full range of TPC2 functions are only beginning to be understood, it has already emerged as an intracellular ion channel of significant pathophysiological relevance, including in a variety of cancer-related functions. This review will summarize developments in our understanding of TPC2 in pathophysiological conditions, with a focus on cancer, and highlight the pre-clinical evidence for targeting TPCs as a novel anti-cancer therapeutic strategy.

## TPC structure and activation

TPCs comprise a family of voltage- and ligand-gated Na^+^/Ca^2+^ ion channels exclusively located in endolysosomes. TPC1 and 3 are voltage-gated channels, whereas TPC2 opens in response to binding phosphatidylinositol-3,5-diphosphate [PI(3,5)P_2_] and nicotinic acid adenine dinucleotide phosphate (NAADP) [6–8]. However, the role of TPC2 in NAADP-mediated Ca^2+^ release has been controversial [9, 10]. In an effort to resolve this controversy, Jha et al. [11] identified regulators of TPC2, and demonstrated that Mg^2+^ can inhibit these NAADP-mediated Ca^2+^ currents, but that when Mg^2+^ is absent, NAADP can activate TPC2, which may account for the conflicting findings.

**Figure 1.**
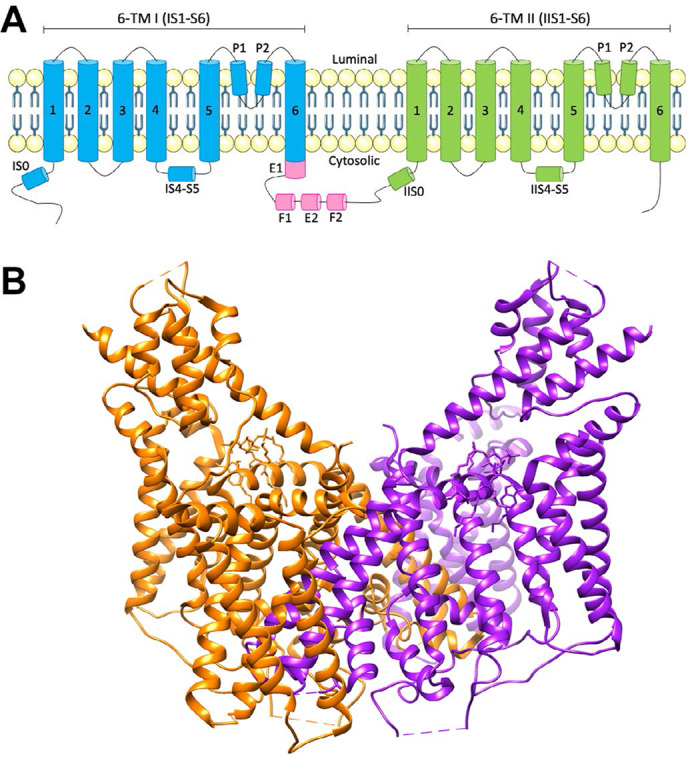
Schematic of TPC2 structure. A) Topology and domain arrangement of a human TPC2 subunit; B) crystal structure of human TPC2. PDB: 6NQ2. Generated using Chimera [17]. The two different protein subunits are shown in orange and purple. E F: EF-hand motifs; P: pore domain; TM: transmembrane

While three distantly related genes (*TPC1*–*3*) have been identified, humans and rodents only express two isoforms, TPC1 and TPC2 [12], encoded by genes in humans located on chromosomes 12 and 11, respectively. TPC2 expression is localized to the late endosome and lysosomal membranes, whereas TPC1 shows a broader distribution throughout the endolysosomal system, particularly in earlier, less acidic endosomal compartments [7, 13]. While the name “TPC” suggests an ion channel with two separate pores, TPCs actually consist of two subunits forming a dimer. Each TPC subunit is comprised of two homologous shaker-like six transmembrane domain repeats (labeled IS1–S6 and IIS1–S6) (Figure 1), with the two-pore loop domains located in IS5–S6 and IIS5–S6 (Figure 1). PI(3,5)P_2_ binds to the first 6 transmembrane domains to activate the channel independently of voltage changes, inducing a structural change at IS6 [14]. However, the binding region of NAADP remains unknown. TPC1 and TPC2 subunits form homo- and hetero-dimers, with four pore domains forming the central channel in a two-fold symmetric arrangement [15, 16].

## TPC interactome

**Figure 2.**
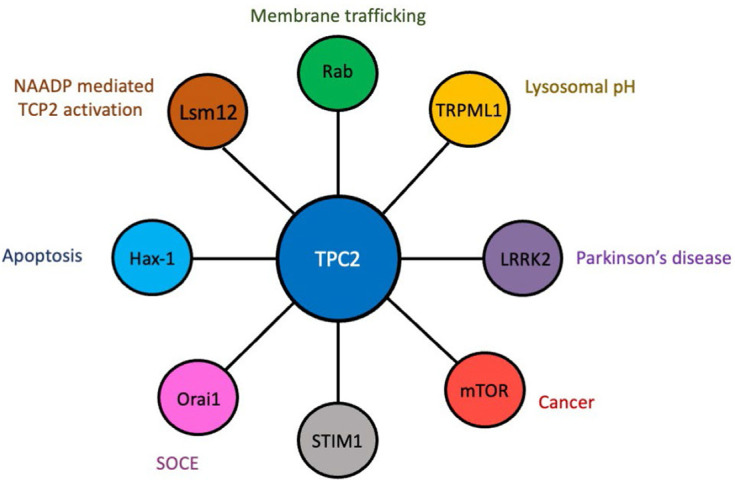
The TPC2 interactome. Diagrammatic representation of proteins that complex with TPC2 and the cellular/physiological processes in which they are known to play a role

While NAADP can endogenously regulate Ca^2+^ release via TPC2, how it does so remains unknown, and it has been suggested that a separate unidentified NAADP-binding protein acts to accessorize TPC2 activation. As a result, the identification of NAADP-TPC2 interactomes is currently an area of great interest (Figure 2), and proteomic characterisation has unsurprisingly revealed that TPC2 complexes with proteins involved in Ca^2+^ homeostasis, trafficking and membrane organisation, including scaffold Rab GTPases, where Rab binding was essential for NAADP-evoked TPC2 Ca^2+^ release [18]. TPC2 can also associate with the transient receptor potential mucolipin 1 (TRPML1) ion channel, but despite this interaction, TRPML1 and TPC2 function as independent ion channels [19]. Although the physiological relevance of this interaction remains unknown, it is most likely related to the control of lysosomal pH. Additionally, TPC2 can associate with the leucine-rich repeat kinase 2 (LRRK2), mutations in which are linked to late-onset Parkinson’s disease [20], and the mammalian target of rapamycin (mTOR), a serine/threonine protein kinase that when hyperactivated leads to increased growth and proliferation [10]. These findings suggest that TPC2 may be involved in the pathological processes of both Parkinson’s disease and cancer. Silencing of endogenous expression of *TPC2*, but not *TPC1*, reduces store-operated Ca^2+^ entry (SOCE), which is modulated through the endoplasmic reticulum Ca^2+^ sensor stromal interaction molecule-1 (STIM1) and plasma membrane Orai1 Ca^2+^ channels, both of which were found to associate with TPC2 [21]. A yeast 2 hybrid assay identified hematopoietic cell-specific protein 1 (HS1)-associated protein X-1 (Hax-1) as a novel binding partner for both TPC1 and TPC2 [22]. Hax-1 has recently been implicated in apoptotic signaling [23], and the TPC/Hax-1 interaction may potentially play a role in modulating apoptosis. A recent study has identified a largely uncharacterized Sm-like protein Lsm12 complexed with NAADP, TPC1, and TPC2 [24], thus potentially identifying the missing accessory protein that has been postulated to exist for NAADP-mediated activation of TPC2. A better understanding of the TPC interactome may potentially lead to the identification of effective pharmacological modulators of TPC-mediated cellular functions, which will have broad implications for a variety of disease processes.

## TPC2 functions

TPC2 plays important roles in various diseases and physiological conditions, including cell proliferation, differentiation, development, autophagy, membrane trafficking, endolysosomal degradation pathway, phagocytosis, angiogenesis, low-density lipoprotein (LDL)-cholesterol trafficking, T cell activation, hormone secretion from the pancreas, and cardiac function [25–34]. Consequently, NAADP/TPC2/Ca^2+^ signaling has been shown to play a critical role in a variety of associated pathophysiological processes, including the life cycle of Ebola virus, human immunodeficiency virus (HIV), Middle East respiratory syndrome coronavirus (MERS-CoV), and severe acute respiratory syndrome coronavirus 2 (SARS-CoV-2), Parkinson’s disease, Alzheimer’s disease, non-alcoholic fatty liver disease, and cardiac dysfunction [28, 31, 35–42], thus highlighting the importance of the endolysosomal system in a variety of pathophysiological processes. Importantly, pharmacological inhibitors of TPC2 are potentially beneficial for the treatment of Ebola virus, SARS-CoV-2, Parkinson’s disease, and Alzheimer’s disease [35, 40, 41, 43], highlighting that TPC2 may be a suitable drug target for a plethora of pathophysiological processes.

**Figure 3.**
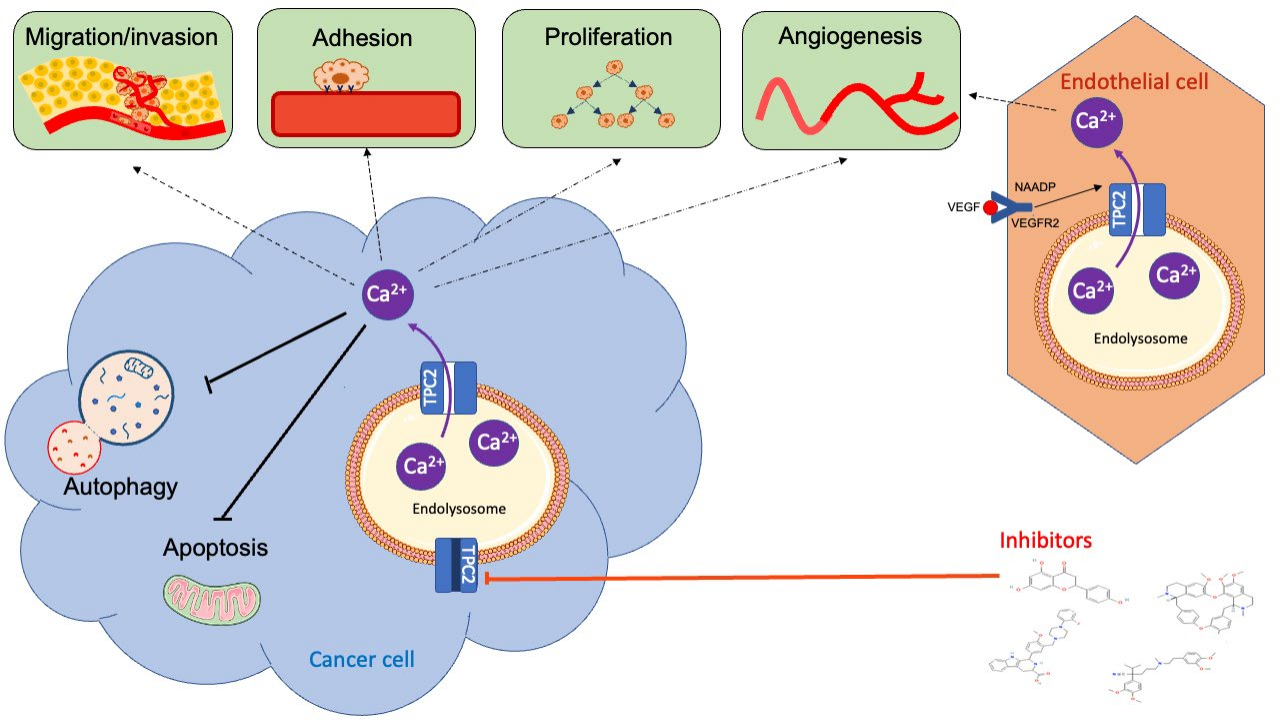
Overview of the role of TPC2 in cancer-related processes. The TPC2/NAADP/Ca^2+^ signaling pathway has been implicated in proliferation, apoptosis, adhesion, invasion, migration, autophagy, and angiogenesis *in vitro* and *in vivo*. Several TPC2 pharmacological inhibitors, including tetrandrine, verapamil, Ned-19 and naringenin, have been demonstrated to inhibit these cancer-related processes *in vitro* and *in vivo*. VEGF: vascular endothelial growth factor; VEGFR2: vascular endothelial growth factor receptor 2

Of particular note, TPC2 is overexpressed or mutated in several cancer types. Additionally, TPC2 has recently been implicated in a variety of cancer-related processes (such as proliferation, adhesion, migration, invasion, angiogenesis, and autophagy) that underpin the key hallmarks of cancer [2] (Figure 3).

## TPC2 function in cancer-related processes

### Cell growth and differentiation

A role for TPC2 in proliferation was first postulated based on the finding that TPC2 function could be regulated by the protein kinases, p38 mitogen activated protein kinase (MAPK), c-jun N-terminal kinase (JNK), and the mTOR complex 1 (mTORC1) [11]. Since then, TPC2 has been directly implicated in controlling cancer cell growth and differentiation in a variety of different cell types. Knockdown of *TPC2* expression in pulmonary artery smooth muscle cells, MNT-1 human melanoma cells, RIL175 murine hepatocellular carcinoma cells, and 4T1 murine breast cancer cells significantly decreased proliferation *in vitro* [29, 44–46], demonstrating that TPC2 can control both normal and cancerous cell proliferation. Additionally, decreasing *TPC2* expression in hepatocellular carcinoma cells reduced glycolysis and respiratory activity *in vitro* [45], potentially accounting for these proliferative effects, and completely abrogated hepatocellular carcinoma tumor growth *in vivo* [45]. Further, knockout of the *TPCN2* gene in murine primary melanoma cells decreased initial proliferation rates by slowing G_2_ phase progression [47]. By contrast, *TPC2* overexpression in 4T1 breast cancer cells did not influence proliferation [29], suggesting that this control of proliferation may not be related to the level of TPC2 expression, but rather to the presence of TPC2. Taken together, these results indicate that TPC2 controls cancer cell proliferation highlighting that it may be a useful target for controlling cancer cell growth.

Changes in *TPC2* expression have been observed at various stages in differentiation in multiple cell types, suggesting that TPC2 may control differentiation. For example, *TPC2* mRNA expression was highest in the first 3 days of differentiation of C2C12 myoblast cells but was reduced thereafter, and TPC2 knockdown inhibited differentiation of these myoblasts [48]. Similarly, *TPC2* mRNA was increased in osteoclast precursor cells upon receptor activator of nuclear factor kappa B (NFκB) ligand (RANKL)-induced osteoclast differentiation, and downregulation of TPC2 suppressed osteoclastogenesis [49]. By contrast, in mouse day 3 embryonic stem cells, TPC2 was markedly decreased during the early stages of neural differentiation but increased at later times during neuronal differentiation [50]. As a result, *TPC2* knockdown accelerated the initial commitment of neural progenitors, but inhibited later neuronal differentiation. Additionally, overexpression of *TPC2* induced cell death and prevented embryonic stem cells from differentiating into early neural lineages [50]. Interestingly, *TPC2* knockdown had no effect on the differentiation of astrocytes or oligodendrocytes [50]. Taken together, these studies demonstrate that the role of TPC2 in promoting differentiation is cell type specific.

### Cancer cell migration, invasion, and metastasis

Emerging evidence has also implicated TPC2 in all the major steps of tumor metastasis, namely adhesion, migration, and invasion. Knockdown of *TPC2* expression in T24 bladder cancer and CHL1 and B16-F10 melanoma cells, and pharmacological inhibition in T24, HUH7 liver cancer and 4T1 breast cancer cells, significantly reduced adhesion [47, 51]. Mechanistically, the knockout of the *TPC2* gene in CHL1 and B16-F10 melanoma cell lines decreased adhesion to collagen type I matrix and was associated with a decrease in α2β1 integrin expression on the plasma membrane [47]. While pharmacological inhibition resulting in reduced adhesion to collagen type I matrix may initially appear to be counterintuitive and to promote metastasis, several studies have shown that metastasis is instead promoted by increased adhesion of mda-9/syntenin protein to collage type I and activation of β1 integrin signaling complexes [52]. Whether decreasing adhesion to collagen via pharmacological inhibition of TPC2 promotes or inhibits metastasis remains to be seen, highlighting that the future application of pharmacological inhibitors of TPC2 needs to be cautiously evaluated to ensure that they do not promote metastasis in patients.

In support of TPC2 having a metastasis promoting role, knockdown of *TPC2* expression in MNT-2 melanoma cells, T24 bladder cancer cells, RIL175 hepatocellular carcinoma, 4T1 breast cancer, Hela cervical cancer and pulmonary artery smooth muscle cells, and pharmacological inhibition in T24, HUH7 and 4T1 breast cancer cells led to decreased migration *in vitro* [29, 44–46, 51]. Mechanistically, the downregulation of TPC2 resulted in accumulation of enlarged acidic vesicles, as general recycling was impaired, which halted β1-integrin trafficking, and prevented leading-edge formation [51], providing further support that pharmacological inhibition of TPC2-mediated inhibition of β1-integrin signaling will prevent, rather than promote, metastasis. By contrast, *TPC2* overexpression in 4T1 breast cancer cells had no effect on migration *in vitro* [29]. Taken together, these studies suggest that TPC2 can control cancer cell migration *in vitro*, however, similarly as to what was observed for proliferation, the presence of TPC2, rather than an increased level of expression, may be the only requirement for the regulation of migration.

TPC2 has also been implicated in invasion in melanoma cells. Knockout of *TPC2* in MNT-1 human melanoma cells significantly reduced invasion *in vitro* [44], providing further support for TPC2 possessing a metastasis promoting role*.* By contrast, knockout of the *TPC2* gene in CHL1 and B16-F10 primary murine melanoma cells increased invasion, with an associated increase in matrix metalloproteinase 9 (MMP-9) expression [47]. These conflicting studies highlight that additional examination of the precise role of TPC2 in cancer, particularly melanoma, is required to identify whether TPC2 promotes or prevents invasion and metastasis.

Taken together, these *in vitro* studies demonstrate the importance of TPC2 in adhesion, migration, and invasion, and suggest that TPC2 may promote metastasis *in vivo*. Indeed, knockdown of *TPC2* expression and pharmacological inhibition in 4T1 orthotopic breast cancer and RIL175 hepatocellular carcinoma mouse xenograft models reduced the formation of lung metastases *in vivo* [45, 51], providing additional evidence that TPC2 is an important enhancer of metastasis *in vivo*, particularly for breast and hepatocellular cancer, and suggest that TPC2 may be a potential therapeutic target for preventing cancer metastasis, at least in breast and hepatocellular cancers. Because the expression levels of *TPC2* seem to be less important than its presence for most cancer-associated functions, further investigation into TPC2 binding partners in specific cancer cell types will ultimately be required to unravel the pathways responsible for the varying effects observed.

### Angiogenesis

Tumor cells are highly dependent on the formation of new blood vessels to sustain their insatiable growth requirements. This pathological process of neoangiogenesis involves stimulation of normally quiescent endothelial cells through their VEGF receptors to induce proliferation, migration, and capillary branch formation. Production of VEGF by tumor cells is the key mediator of this process, but VEGF also affects immune cells in the tumor micro-environment as well as providing an autocrine pro-malignancy signal that promotes epithelial-mesenchymal transition of the cancer cells [53]. VEGF inhibitors initially appeared promising as anti-cancer therapies but were ultimately overcome by tumor cells that developed compensatory ways to survive [54]. The discovery that signaling through the VEGFR2 receptor subtype involves NAADP and TPC2-dependent lysosomal Ca^2+^ release [33] offers alternative strategies for targeting this pathway. In these studies, TPC2, but not TPC1, knockdown in endothelial cells inhibited VEGF-induced Ca^2+^ release and angiogenesis *in vitro* [33]. Further, pharmacological inhibition of TPC2 decreased angiogenesis in glioma and breast cancer models *in vivo* [55–57]. Additionally, *Tpcn2*^−/−^ mice or pharmacological inhibition of TPC2 inhibited vascularisation of VEGF-containing matrigel plugs *in vivo* [33, 58]. TPC2 has also been implicated in controlling VEGF-dependent neovascularization in a mouse model of age-related macular degeneration [59], demonstrating that these angiogenic pathways are likely to be a normal response to VEGF-VEGFR2 signaling in endothelial cells. In contrast, autocrine VEGF signaling in cancer cells is expected to be dysregulated through alterations in TPC2 function. VEGFR2 is expressed by a multitude of cancers, ranging from solid tumors (such as brain, gynaecological, gastrointestinal, lung) to those of the haematopoietic system (multiple myeloma, myeloid leukaemias) [3]. Thus, pharmacological targeting of TPC2 will have many effects on many cell types, with the potential to alter both the tumor and its microenvironment.

### Autophagy

Autophagy is a lysosomal dependent mechanism used to naturally rid the cell of dysfunctional or redundant elements and is a critical regulator of cellular homeostasis. In extreme cases of starvation, a cell will use autophagy as a source of energy to maintain survival, and defects in this pathway have been associated with numerous human diseases, including cancer [60]. However, autophagy plays a dual role in cancer, where it can play a tumor suppressive role in normal tissue but promote tumorigenesis in cancer tissue, by increasing proliferation, angiogenesis, metastasis, and decreasing apoptosis [61]. The lysosomal location of TPC2 implies involvement in autophagic pathways, but this is enigmatic and there is debate as to whether TPC2 promotes or hampers autophagy.

Several studies have indicated that TPC2 is a positive regulator of autophagic flux. Overexpression of *TPC2* in astrocytes increased the expression of the autophagic markers LC3-II and beclin-1, as well as acidic vesicular organelle formation [62]. Conversely, expression of a dominant-negative *TPC2* construct led to decreased LC3-II levels in these astrocytes, and knockdown of *TPC2* expression decreased autophagy in astrocytes in an mTOR-independent manner [63]. Furthermore, in cardiomyocytes, starvation induced a significant increase in TPC1 and TPC2 expression that paralleled increased autophagy [64]. Additionally, silencing of *TPC2* alone or in combination with *TPC1* in nutrient-rich conditions induced autophagy, whereas under starvation, silencing of either *TPC1* or *TPC2* induced an autophagic block [64]. This study suggests that TPC2 is mainly required for basal autophagy, while both TPC1 and TPC2 are required for starvation-induced autophagy. Additionally, one of the identified TPC2 binding partners, LRRK2, is a regulator of autophagy, involving activation of the calcium/calmodulin-stimulated protein kinase kinase β (CaMKKβ)/adenosine monophosphate (AMP)-activated protein kinase (AMPK) pathway [20]. These LRRK2-mediated effects on autophagy could be completely abrogated by the expression of a dominant-negative, but not wild-type, TPC2 construct [20], identifying a potential functional role for the LRRK2/TPC2 interaction.

By contrast, there is also evidence that TPC2 can act as a negative regulator of autophagic flux. Overexpression of TPC2, but not an inactive mutant form of TPC2, in Hela cervical cancer and 4T1 breast cancer cells decreased autophagosomal-lysosomal fusion, resulting in the accumulation of autophagosomes [29, 65]. Conversely, *TPC2* knockdown in mouse embryonic stem cells promoted autophagosomal-lysosomal fusion [65], and autophagy-related gene 5 (*ATG5*) knockdown, but not mTOR activity inhibition, abolished the TPC2-induced accumulation of autophagosomes [65]. Further, skeletal muscles from *Tpcn2*^−/−^ mice exhibited enhanced autophagy flux characterized by increased accumulation of autophagosomes upon starvation and treatment with the microtubule inhibitor, colchicine [66]. Taken together, the studies demonstrating a positive regulation of autophagy effect for TPC2 have largely involved examination in normal cells, whereas the studies showing a negative regulation of autophagy mostly investigated cancer or embryonic stem cells, suggesting that the cell type will influence the TPC2-mediated effect on autophagy.

Further controversy surrounds the mechanisms by which TPC2 may mediate autophagy. While the TPC-mediated effects in astrocytes occurred in an mTOR-independent manner [63], pharmacological inhibition of TPC2 in HGC-27 gastric cancer, T24 and 5637 bladder cancer cells and MDA-MB-231 breast cancer cells induced autophagy by inhibiting mTOR signaling pathway [67–69], suggesting that TPC2- mediated positive regulation of autophagy in normal cells may occur in an mTOR-independent manner, but that TPC-mediated negative regulation of autophagy may occur in an mTOR-dependent manner.

## TPC2 alterations and expression in cancer

As TPC2 has been implicated in several cancer related processes, it raises the question as to whether abnormalities in TPC2 gene coding or protein expression may be observed in cancer. Inherited polymorphisms in *TPC2* have been identified as predisposition factors for a range of cancer types [70–72], most notably melanoma due to the role of TPC2 in controlling pigmentation through regulation of melanosomes [73]. However, acquired abnormalities within the *TPC2* coding region appear restricted to rare instances of gene fusions in breast cancer and uterine leiomyomas [74, 75]. The chromosomal region harbouring *TPC2* (11q13.2) is commonly amplified in cancer [76], and overexpression of the *TPC2* gene has been identified as the potential driver of this amplification by providing growth advantage in oral squamous cell carcinoma [77, 78]. Hepatocellular carcinoma patient samples show highly positive staining for TPC2, and the majority of breast cancer and non-tumorigenic breast samples express TPC2 [51, 79]. Additionally, increased expression of TPC2 has been observed in cell lines derived from blood, liver and bladder cancer relative to MDA-MB-231 breast cancer cells [51].

*TPC2* expression is also a potential prognostic biomarker for several types of solid tumors. *TPC2* was part of a gene signature for biomarkers of lymph node metastasis in oral squamous cell carcinoma, recurrence in prostate cancer, and decreased overall survival in bladder cancer [77, 80, 81], and has been associated with increased risk of melanoma [82]. Taken together, these studies indicate that *TPC2* is highly expressed in hepatocellular carcinoma, oral squamous cell carcinoma and blood cancers, however, whether this expression is observed in additional cancer types, remains to be seen. Additionally, increased TPC2 expression may be a biomarker for poor prognosis for several types of cancer, including oral, prostate and bladder cancers. These negative effects on patient prognosis are most likely due to the central role of TPC2 in regulating cancer cell proliferation and metastasis and highlight that inhibiting TPC2 activity is likely to be an anti-cancer therapeutic strategy for a range of cancer types.

## Pharmacological inhibition of TPC as an anti-cancer strategy

Due to the importance of TPC2 in controlling cancer-related functions (Figure 3), its suitability as an anti-cancer target is beginning to be appreciated. Several pharmacological inhibitors of TPC2 have been identified (Table 1) and their pre-clinical efficacy in a range of cancer types has recently begun to be explored (Table 2).

**Table 1.** Summary of pharmacological inhibitors of TPC2 that have been pre-clinically evaluated in cancer

**Name**	**Class**	**Structure**
Naringenin	Flavonoid	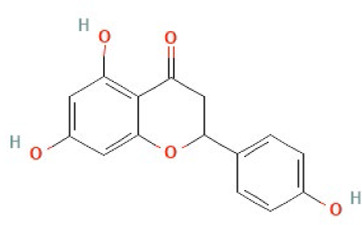
Tetrandrine	Ca^2+^ channel blocker	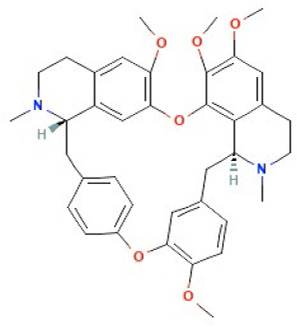
Ned-19	NAADP-antagonist	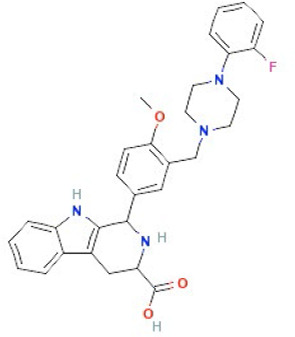
Verapamil	Ca^2+^ channel blocker	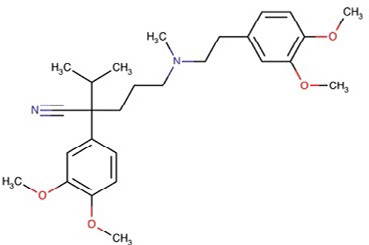

Structures obtained from PubChem [83].

*Note.* Structure image of Naringenin is reprinted from https://pubchem.ncbi.nlm.nih.gov/compound/932#section=2D-Structure ; structure image of Tetrandrine is reprinted from https://pubchem.ncbi.nlm.nih.gov/compound/73078#section=2D-Structure; structure image of Ned-19 is reprint from https://pubchem.ncbi.nlm.nih.gov/compound/3978027#section=2D-Structure; structure image of Verapamil is adapted from https://pubchem.ncbi.nlm.nih.gov/compound/2520#section=2D-Structure

### Naringenin

Naringenin, a natural flavonoid found in citrus and tomatoes, is one of the main flavonoids present in the human diet [84]. Naringenin has a wide range of potential applications, including anti-viral, anti-ageing, anti-inflammatory, lipid lowering, anti-microbial, blood thinning and cardioprotective properties [84]. Anecdotally, higher serum concentrations of flavonoids, including naringenin, are significantly associated with lower breast, lung, and gastric cancer risk [85–89], suggesting that naringenin may possess anti-cancer properties. Mechanistically, naringenin can inhibit TPC2 and dampen the NAADP-stimulated Ca^2+^ response [58], and as such has been explored as a TPC2 inhibitor. However, naringenin also has other non-TPC2 related actions, and is predicted to act as an estrogen receptor α (ERα) agonist [90], so its effects cannot be attributed solely to TPC2 inhibition.

Naringenin exhibits anti-proliferative and pro-apoptotic effects in glioma, melanoma, breast, colorectal, hepatic, prostate, lung, and pancreatic cancer *in vitro* [91–114], and in glioma, melanoma, breast, colorectal, hepatic, and lung cancer xenografts *in vivo* [91, 96–98, 115–121] (Table 2). By contrast, HCT116, HT29, and T84 colorectal cancer cells were only slightly sensitive to naringenin treatment *in vitro* but combined with 5-fluorouracil synergistically enhanced cell death [122]. Additionally, combining naringenin with paclitaxel synergistically increased cytotoxicity in prostate cancer cells *in vitro* [123], and when combined with histone deacetylase inhibitors, synergistically suppressed neuroblastoma tumor progression *in vivo* [124]. Taken together, these studies suggest that naringenin can act as a chemosensitiser. Indeed, naringenin has been demonstrated to alleviate multidrug resistance against gemcitabine and anthracyclines in a range of cancer types [106, 125]. It is likely that naringenin will be most beneficial clinically in a combinatorial setting to alleviate multi-drug resistance in advanced cancers.

**Table 2.** Summary of pre-clinical evaluation of pharmacological inhibitors of TPC2, including proposed down-stream mechanisms

**Cancer**	**Cell line(s)**	**Effects**				**Mechanism**	**Ref**
		**Growth**	**Cell death**	**Migration & invasion**	**Growth *in vivo***		
Naringenin							
Glioma	C6	-	-	-	↓	ROS, cyclin D1, NFκB, CDK4	[118]
	C6	-	-	-	↓	PI3K, PKB	[119]
	U-118MG	-	↑	-	-	-	[108]
	GBM8401	None	↑	↓	-	MMP	[109]
Melanoma	B16F10	↓	↑	-	↓ (lung mets)	Transglutaminase	[110]
	B16F10	↓	↑	-	-	-	[111]
	B16F10, SK- MEL-28	↓	↑	↓	-	ERK1/2, JNK, MAPK, PARP, caspase	[112]
	B16F10	-	-	-	↓	TGFβ-Smad-MMP2	[120]
Breast cancer	MDA-MB-435	↓	-	-	↓ (delayed tumor growth; DMBA rat)	-	[91]
	4T1	None	-	-	↓ (lung mets)	IFN-γ, IL-2	[126]
	MCF-7, T47D, MDA-MB-231	↓	↑	-	-	Caspase, p38	[92]
	MDA-MB-231, MDA-MB-468	-	↑	-	-	-	[93]
	HTB26, HTB132	↓	↑	-	-	Cyclins, caspases, PI3K/Akt pathway, NFκB	[94]
	SKBR3, MDA- MB-231	↓	↑	-	-	HER2	[95]
	4T1	-	-	-	↓ (lung mets)	TGFβ	[127]
	E0771	↓	↑	-	↓ (delayed tumor growth)	AMPK, cyclin D1	[96]
	MDA-MB-231	↓	↑	↓	↓	Mitochondria, NFκB, biotransformation enzymes	[97]
	MCF-7, T47D	-	↑	-	↓	Aromatase	[98]
Colorectal cancer	Azoxymethane rat model	-	-	-	↓	-	[115]
	HCT116	↓	-	-	-	Cell cycle regulatory protein expression	[99]
	SW1116, SW837	↓	↑	-	-	Cyclins, caspases, PI3K/Akt pathway, NFκB	[94]
	HCT116, SW480	↓	-	-	-	Cyclin D1, p38	[100]
	HT29	↓	↑	-	-	Cell cycle and death pathways	[101]
	Caco-2	↓	-	-	-	ROS	[102]
	HCT116, HT29, T84	None	None	-	-	MAPK	[122]
Hepatic carcinoma	HepG2	↓	↑	-	-	P53, caspase	[103]
	NDEA-induced rat model	-	-	-	↓	PCNA, Bcl-2, NFκB, VEGF, MMP	[116]
	NDEA-induced rat model	-	-	-	↓	Antioxidant	[117]
Prostate cancer	PC3, LnCaP	↓	↑	↓	-	ROS, mitochondria	[104]
	PC3	-	-	↓	-	EMT, uPA activity	[128]
	Mat-LyLu	↓	-	↓	-	SCN9A	[105]
Pancreatic cancer	ASPC-1, PANC-1	-	↑	↓	-	EMT	[106]
	SNU-213	-	↑	-	-	ROS	[107]
Lung cancer	NRG mice model	-	-	-	↓	CYP1A1, NFκB, PCNA	[121]
	A549	↓	-	↓	-	MMP-2/9, Akt	[113]
	A549	↓	↑	↓	-	Caspase, MMP	[114]
	LLC	-	-	-	↓	TGFβ-Smad-MMP2	[120]
Tetrandrine							
AML	U937	↓	↑	-	-	Caspase, JNK, PKC-δ	[129]
	NB4	↓	-	-	↓	ROS, Notch-1	[130]
	K562, 6133 MPL^W515L^	↓	↑	-	-	P21, p27, ROS, Notch-1 signaling	[131]
	HL60, K562, U937, THP-1	↓	↑	-	↓	ROS, c-myc	[132]
Glioma	RT-2	↓	↑	-	↓	-	[55]
	U-87	↓	↑	↓	-	ADAM17, PI3K/Akt signaling pathway	[133]
	U-87, U251, SWO- 38	↓	↑	-	↓	STAT3	[56]
	U-87, U251	↓ (Neurosphere formation)	↑	-	-	β-catenin, PARP, Bcl-2	[134]
	U-87, U251	↓	-	-	-	ERK	[135]
	GBM 8401	-	-	↓	-	MMP-2/9, NFκB, Akt, EGFR, E/N-cadherin	[136]
Neuroblastoma	Neuro2a	↓	↑	-	-	ROS	[137]
	SHSY5Y	-	↑	-	-	-	[138]
Osteosarcoma	U-20S, MG-63	-	↑	-	-	Apaf-1, Bid, Bax, Bcl-2	[139]
	143B	↓	↑	-	↓	PTEN, PCNA	[140]
Nasopharyngeal carcinoma	CNE	-	↑	-	-	Bax/Bcl-2	[141]
	NPC-TW076	↓	↑	-	-	Endoplasmic reticulum stress	[142]
Lung cancer	A549	↓	↑	-	-	Akt, ERK	[143]
	A549	↓	↑	-	-	VEGF/HIF-1/ICAM-1	[144]
Oral cancer	SAS	-	↑	-	-	PARP, caspase	[145]
	HSC-3	-	↑	-	-	PARP, caspase, beclin-1/LC3-1/II signaling	[146]
	CAL27	-	↑	-	-	ROS, caspase, Beclin-1 signaling	[147]
Gastric cancer	BGC-823	-	↑	-	-	Caspase/mitochondria- mediated	[148]
	HGC-27	-	↑	-	-	PARP, caspase, Beclin-1/LC3-II/p62, Akt/mTOR	[67]
Prostate cancer	DU145, PC3	↓	↑	-	-	ROS, JNK1/2	[149]
	DU145, PC3	↓	↑	↓	-	PARP, PI3K/Akt	[150]
	DU145, PC3	-	-	↓	-	Akt/mTOR/MMP-9 signaling	[151]
Bladder cancer	5637, T24	-	↑	-	-	Caspase/mitochondria- mediated	[152]
	5637, T24	-	-	↓	-	Inducing MET through downregulation of Gli-1	[153]
Breast cancer	SUM-149, SUM-159, sphere (patient sample)	↓	-	-	-	-	[154]
	4T1	-	-	-	↓ (lung mets)	ERK, NFκB, VEGF, HIF-1α, integrin β5 and ICAM-1	[57]
	MCF-7	-	↓	-	-	PKCα, caspase	[155]
	MDA-MB-231	↓	↑	-	-	Beclin-1/LC3-I/LC3-II and PI3K/Akt/mTOR signaling pathways	[68]
	MDA-MB-231	-	↑	-	↓	Bcl-2, Bax, PARP, caspase	[156]
Renal cell carcinoma	786-O, 769-P, ACHN	↓	↑	-	-	Caspase, p21 and p27	[157]
	786-O, 769-P	-	-	↓	-	MMP-9, PI3K/Akt, NFκB	[158]
Hepatic carcinoma	HepG2	↓	↑	-	-	Caspase	[159]
	HepG2	↓	↑	-	-	Caspase	[160]
	Hep G2, Hep 3B, PLC/PRF/5	↓	-	-	-	-	[161]
	HepG2, Huh7	-	↑	-	↓	ROS, Akt	[162]
	Huh-7	-	-	-	↓	ROS, ERK	[163]
	Huh-7	↓	↑	-	-	Caspase, PARP	[164]
	Huh7, SMMC- 7721, HepG2, PLC/PRF/5, MHCC97H, SK- Hep-1, SNU398	↓	-	-	-	CaMKII	[165]
Colorectal cancer	CT-26	-	↑	-	↓ (lung mets)	-	[166]
	HT-29	↓	↑	-	-	PI3K/Akt/GSK3β, PARP	[167]
	CT-26	-	↑	-	↓	P38 MAPK	[168]
	SW480, HCT116	↓	↑	↓	↓	Wnt/β-catenin,	[169]
	LoVo	↓	↑	-	↓	IGFBP-5, Wnt/β-catenin	[170]
	HCT116	↓	↑	↓	-	MMP-2, EMT	[171]
Cervical cancer	SiHa	↓	↑	↓	↓	Caspase, MMP-2, MMP-9	[172]
Gallbladder cancer	SGC-996	↓	↑	-	-	Caspase, PARP, mitochondria	[173]
Pancreatic cancer	PaCa	↓	-	-	↓	P21, p27, cyclin D	[174]
Ovarian cancer	OVCAR-3, A2780	↓	↑	-	↓	Wnt, E-cadherin, cyclin D, c-myc	[175]
Ned-19							
Bladder cancer	T24	-	-	↓	-	Endocytic recycling	[51]
Hepatic carcinoma	Huh7	-	-	↓	-	Endocytic recycling	[51]
Breast cancer	4T1	-	-	↓	↓	Endocytic recycling	[51]
Melanoma	B16	↓	↑	↓	↓	VEGF	[176]
Colorectal cancer	Patient samples	↓	-	-	-	ERK, Akt	[177]
Verapamil							
Breast cancer	ZR-751A	↑	-	-	-	-	[178]
Colorectal cancer	LoVo	↑	-	-	-	-	[178]
AML	Patient samples	↓	-	-	-	-	[179]
	Patient samples	↓	-	-	-	-	[180]
CML	Patient samples	None	-	-	-	-	[180]

ADAM17: ADAM metallopeptidase domain 17; AML: acute myeloid leukaemia; Bcl-2: B-cell lymphoma-2; CaMKII: calcium/calmodulin-stimulated protein kinase II; CDK4: cyclin-dependent kinase 4; CML: chronic myeloid leukaemia; DMBA: 7,12-dimethylbenz[a]anthracene; EGFR: epidermal growth factor receptor; EMT: epithelial to mesenchymal transition; ERK1: extracellular signal-regulated kinase 1; GSK3β: glycogen synthase kinase 3 β; HER2: human epidermal growth factor receptor 2; HIF-1: hypoxia inducible factor 1; ICAM-1: intracellular adhesion molecule 1; IFN: interferon; IGFBP: insulin-like growth factor binding protein; IL-2: interleukin-2; MET: mesenchymal to epithelial transition; mets: metastases; NDEA: *N*-nitrosodiethylamine; PARP: polyadenosine-diphosphate-ribose polymerase; PCNA: proliferating cell nuclear antigen; PKB: protein kinase B; PI3K: phosphoinositide-3-kinase; PTEN: phosphatase and tensin homolog; Ref: reference; ROS: reactive oxygen species; SCN9A: sodium voltage-gated channel α subunit 9; STAT3: signal transducer and activator of transcription 3; TGFβ: transforming growth factor β; uPA: urokinase type plasminogen activator; ↓: decreased effects; ↑: increased or induced effects; -: not examined

Further, naringenin inhibits migration of glioma, melanoma, breast, prostate pancreatic, and lung cancer cells *in vitro* [97, 104–106, 109, 112–114, 128] (Table 2), suggesting that naringenin may act as an anti-metastatic agent, and that pharmacologically inhibiting TPC2 will inhibit, rather than promote metastasis. Indeed, naringenin can decrease lung metastases in 4T1 breast cancer and B16F10 melanoma xenografts *in vivo* [110, 126, 127]. Anti-cancer agents that are effective against metastases are rare, and these pre-clinical findings highlight that naringenin, and inhibiting TPC2, may be a promising avenue for the treatment of metastatic cancers.

Several studies have investigated the mechanisms underlying naringenin-mediated anti-cancer effects and have identified a variety of mechanisms (Table 2), including immunomodulation, NFκB-signaling, ROS-mediated induction of apoptosis, PI3K/Akt pathway, and TGFβ-mediated signaling pathways. Many of these pathways are known to be activated in cancer cells, thus providing additional support for naringenin being a suitable anti-cancer agent.

In an attempt to improve the effectiveness of naringenin, derivatives modified at position 7 have been developed. Initial examination has shown that HCT116 colon cancer cells were more sensitive to these derivatives than to naringenin [181], however, examination in additional clinically relevant laboratory models is required to ascertain whether cancer cells in general are more sensitive to these derivatives. Further, additional flavonoids from a Southeast Asian plant extract (*Dalbergia parviflora*) block TPC2 activity in melanoma cells and inhibit melanoma cell proliferation, migration, and invasion *in vitro* [44]. Further evaluation of these flavonoids is required; however, they may be an additional avenue of promising research.

Despite the abundance of pre-clinical evidence indicating that naringenin is a potent anti-cancer agent with low toxicity (Table 2), this has not been investigated clinically in cancer. Importantly, bioavailability of ingested natural compounds may be lower than required to exert an anti-cancer effect at a cellular level. However, clinical trials evaluating the safety and pharmacokinetics of naringenin in healthy adults have shown that naringenin ingestion had no related adverse events or alterations in blood safety markers [182]. Most promising, maximal blood concentrations of 48.45 ± 7.88 μmol/L were achieved within 4 h, with a half-life of 2–3 h. These concentrations are compatible with those required for inhibitory effects against cancer cells *in vitro*, with reported IC_50_ values ranging from 2.2–178 μmol/L for most cell lines [183]. Future efforts should involve focusing on clinical trials of naringenin, both alone and as a chemosensitizing agent, in advanced cancers, particularly breast cancers.

### Tetrandrine

Tetrandrine is isolated from the plant *Stephania tetrandra* and belongs to the class of bisbenzylisoquinoline alkaloids [184]. Tetrandrine has been used as a traditional medicine in China for decades to treat patients with autoimmune and inflammatory pulmonary disease, silicosis, cardiovascular diseases, and hypertension [185]. Tetrandrine acts as a Ca^2+^ channel blocker, and recently, tetrandrine was shown to block TPC2 currents elicited by both NAADP and PI(3,5)P_2_ [35]. Additionally, tetrandrine inhibits the downstream regulator CaMKII [165], a kinase that has been implicated in cancer progression and metastasis [1, 186, 187]. This raises the question as to whether tetrandrine is blocking TPC2 itself or inhibiting downstream Ca^2+^-signaling pathways. Tetrandrine can also suppress another Ca^2+^-mediated process, specifically the nucleotide-binding domain, leucine-rich-repeat-containing family, pyrin domain-containing 3 (NLRP3) inflammasome activation via Sirt-1 [188, 189]. Taken together, these studies highlight that tetrandrine is not a specific TPC2 inhibitor, but rather inhibits Ca^2+^-signaling related processes. Nevertheless, emerging evidence indicates that tetrandrine possesses anti-cancer efficacy against a range of cancer types (Table 2).

Tetrandrine displayed anti-proliferative and cytotoxic effects as a monotherapy against AML, glioma, neuroblastoma, osteosarcoma, nasopharyngeal, lung, oral, gastric, prostate, bladder, breast, renal cell, hepatocellular, colorectal, gallbladder, pancreatic, and ovarian cancer cells *in vitro* [55, 56, 67, 68, 129–135, 137–146, 148–150, 152, 154–157, 160–162, 164, 165, 167–175 ], and decreased tumor growth in glioma, osteosarcoma, AML, breast, hepatocellular, cervical, pancreatic, colorectal, and ovarian cancer xenograft models *in vivo* [55, 56, 130, 132, 140, 147, 156, 159, 162, 163, 168, 169, 172, 174, 175 ] (Table 2). By contrast, despite nasopharyngeal cancer cells exhibiting sensitivity to tetrandrine *in vitro*, nasopharyngeal carcinoma xenografts were not sensitive to tetrandrine as a monotherapy *in vivo*, however tetrandrine was able to sensitise these tumors to irradiation [190], suggesting that it may act as a radiosensitising agent. Further, tetrandrine can also synergistically enhance the effects of numerous chemotherapeutics, including 5-flourouracil, imatinib, paclitaxel, vincristine, daunorubicin, cisplatin, sorafenib, arsenic trioxide, chloroquine, and the protein kinase inhibitor H89 both *in vitro* and *in vivo* [169, 175, 191–198 ], indicating that like naringenin, tetrandrine is also a chemosensitizing agent.

There is also substantial pre-clinical evidence that tetrandrine may be effective against metastatic disease. Tetrandrine treatment decreases invasion and migration of a range of cancer types *in vitro* [51, 133, 134, 136, 150, 151, 153, 158, 169, 171, 172 ], and importantly, the formation of lung metastases in an orthotopic 4T1 breast cancer and an intravenous CT-26 colorectal cancer model *in vivo* [51, 57, 166] (Table 2), indicating that tetrandrine may be an effective agent for the treatment of metastatic disease.

Several studies have investigated the mechanisms underlying tetrandrine-mediated anti-cancer effects (Table 2). Although a variety of different mechanisms were identified, several were repeatedly observed, including ROS-mediated induction of apoptosis, PI3K/Akt/mTOR signaling, and caspase activation, suggesting that these may underpin the anti-proliferative and pro-apoptotic tetrandrine-mediated effects observed in a variety of cancer types.

Taken together, these studies highlight that tetrandrine is an interesting drug candidate for the treatment of cancer as a monotherapy and in combination with a variety of chemotherapeutics. While a large amount of pre-clinical evidence demonstrated that tetrandrine is effective in both *in vitro* and *in vivo* cancer models (Table 2), the majority of these studies were performed in two-dimensional cell culture models in only a single cancer cell line. Not all promising *in vitro* effects will translate into *in vivo* or clinical effects, therefore further examination in clinically relevant *in vivo* models is required before future clinical examination is performed. Additionally, it is likely that tetrandrine will be more beneficial clinically when combined with existing chemotherapeutics.

Nevertheless, a clinical trial examining the combination of intravenous tetrandrine with daunorubicin, etoposide and cytarabine for the treatment of refractory and relapsed AML showed that this combination was relatively well tolerated. Forty-two percent of patients achieved complete remission or restored chronic phase, 23% achieved a partial response, and 34% failed therapy [199], this provides the first evidence that tetrandrine may be a suitable therapy for the treatment of cancer. An additional clinical trial examined tetrandrine in combination with gemcitabine and cisplatin for patients with advanced non-small cell lung cancer. While the addition of tetrandrine to this regimen did not significantly improve the objective response rate, it did improve quality of life scores and mitigated adverse reactions to the chemotherapy [200], providing support for tetrandrine as a chemosensitiser.

Despite these promising results, several animal models have identified that tetrandrine can induce substantial liver and lung damage [201–203], which has raised concerns as to the use of tetrandrine in cancer patients. As such, analogues of tetrandrine, with lower toxicity profiles are under development [184]. Several of these analogues (RMS1–2, RMS4, RMS7–8) can inhibit proliferation and in some cases (RMS1, RMS4, RMS8) induce apoptosis of leukaemia cells that are resistant to a variety of chemotherapeutic drugs [184]. An additional analogue (H1) decreases proliferation and clonogenicity of colorectal, non-small cell lung cancer cells, and doxorubicin-resistant breast cancer cells *in vitro* [204–206]. Tetrandrine analogues that are potent TPC2 specific inhibitors have been identified by screening a library of synthesised benzyltetrahydroisoquinoline derivatives. One of these analogues, SG-094, suppresses tumor growth of RIL175 hepatocellular carcinoma xenografts *in vivo* [45]. While these initial results are promising, examination in additional more clinically relevant laboratory models is required.

### Ned-19

Ned-19 is a selective membrane-permeable non-competitive NAADP antagonist [207], which likely inhibits TPCs in an indirect manner [208]. Ned-19 has primarily been used as an experimental tool to study the functions of TPCs, however, several studies have demonstrated pre-clinical efficacy for Ned-19 (Table 2). Ned-19 treatment decreased melanoma and colorectal cancer cell proliferation and induced apoptosis in B16 melanoma cells *in vitro* [176, 177]. These studies further highlight the role of TPC2 in controlling important cancer-related functions and provide further evidence for the suitability of TPC2 as a potential anti-cancer target.

Further evidence indicates that Ned-19 may be a potential inhibitor of metastasis. Importantly, Ned-19 decreased migration of melanoma, hepatic, bladder, and breast cancer cells *in vitro* [51, 177], and decreased the formation of lung metastases in 4T1 breast cancer and B16 melanoma xenograft models *in vivo* [51, 176]. While these results are promising, additional examination of Ned-19 is required before its clinical utility can be examined.

### Verapamil

Verapamil is a Ca^2+^-channel blocker [209], used clinically to manage angina, arrhythmia and hypertension. Verapamil has also been shown to block NAADP-induced Ca^2+^ release [210], and hence antagonise TPCs. Based on the pre-clinical evidence for the other TPC inhibitors, it would be expected that verapamil also possesses anti-cancer properties. However, controversy exists concerning the potential association between Ca^2+^-channel blockers, particularly verapamil, and increased risk for the development of breast cancer [211]. Indeed, verapamil has a growth stimulatory effect on breast cancer cells *in vitro* [178], suggesting that it may indeed act as a tumor promoter for breast cancers. By contrast, verapamil inhibited proliferation *ex vivo* of tumor cells collected from AML, but not CML patients in blast phase [179, 180]. Taken together, these studies highlight that verapamil exhibits dramatically different effects in different cell types, emphasising that a better understanding of the signaling pathways altered by verapamil in different cancer types is required.

Despite these conflicting results on cancer cell proliferation, verapamil has been widely established to act as a chemosensitiser, by inhibiting the function of p-glycoprotein [212–214]. While *in vitro* evidence spanning several decades indicates that verapamil can sensitise a range of cancer cells to treatment with chemotherapeutics, including doxorubicin, daunorubicin, vincristine, paclitaxel, mitomycin and amlodipine [215–221], this efficacy has not always translated into improved survival benefit or tumor reduction in *in vivo* models. For example, verapamil + doxorubicin combination treatment did not increase survival or reduce metastatic burden in a 4T1-resistant breast cancer xenograft mouse model *in vivo* [215]. Additionally, orally administered verapamil significantly increased doxorubicin toxicity in mice [222]. Further, intravenous administration of verapamil in combination with doxorubicin and vincristine in patients with advanced and refractory breast cancer potentiated neurotoxicity and haemotoxicity [223]. By contrast, arterial infusion of verapamil alone and in combination with chemotherapeutics has been demonstrated to improve medical imaging parameters without increasing toxicity in primary liver cancer patients [224]. Taken together, these pre-clinical and clinical studies indicate that the route of administration of verapamil can influence the toxicity and chemosensitizing activity, and that arterial infusion of verapamil is the optimal clinical administration method. In support of this, targeted arterial perfusion of verapamil and chemotherapeutics (platinum therapy in combination with paclitaxel, docetaxel, 5-fluorouracil, and paclitaxel + gemcitabine + 5-fluorouracil) in advanced lung cancer patients [225] was clinically effective, where 85% of patients achieved a complete or partial remission, and all side-effects experienced with the combinatorial treatments resolved quickly [225]. In addition to being well tolerated, combining arterial verapamil with platinum and 5-fluorouracil and an anthracycline, or with docetaxel and anthracycline in advanced gastric cancer patients, significantly improved the efficacy over chemotherapy alone, and also improved progression-free and overall survival rates compared to chemotherapy alone [226]. These clinical studies identify that verapamil is particularly amenable to combination with platinum agents and also indicates that the route of administration of verapamil may impact its toxicity profile.

## Conclusion

TPC2 is an attractive anti-cancer target as it is expressed in a range of cancer types and is vital in the control of cancer cell proliferation, survival, metastasis, and angiogenesis. Several drugs that inhibit TPC2, including the naturally occurring naringenin and tetrandrine, have been identified which demonstrate pre-clinical anti-cancer efficacy, including reducing metastatic burden *in vivo*. These agents have all been shown to act as chemosensitizing agents, suggesting that they will be most beneficial clinically when used in combination with existing chemotherapeutics. Indeed, several clinical studies have shown that tetrandrine and verapamil can potentiate the effects of chemotherapeutics in advanced cancer patients. However, both of these drugs can induce toxicity, most likely due to their Ca^2+^-channel blocking effects, so different routes of administration and analogues with improved safety profiles are being examined. Overall, a large body of evidence indicates that TPC2 is an attractive anti-cancer target that warrants further investigation.

## Abbreviations

AML: acute myeloid leukaemia
Hax-1: hematopoietic cell-specific protein 1-associated protein X-1
JNK: c-jun N-terminal kinase
LRRK2: leucine-rich repeat kinase 2
MAPK: mitogen activated protein kinase
MMP-9: matrix metalloproteinase 9
mTOR: mammalian target of rapamycin
NAADP: nicotinic acid adenine dinucleotide phosphate
NFκB: nuclear factor kappa B
PI(3,5)P_2_: phosphatidylinositol-3,5-diphosphate
PI3K: phosphoinositide-3-kinase
ROS: reactive oxygen species
TGFβ: transforming growth factor β
TPCs: two-pore channels
TRPML1: transient receptor potential mucolipin 1
VEGF: vascular endothelial growth factor
VEGFR2: vascular endothelial growth factor receptor 2

## Declarations

### Copyright

© The Author(s) 2022.
